# Effects of Selenium Nanoparticles Combined With Radiotherapy on Lung Cancer Cells

**DOI:** 10.3389/fbioe.2020.598997

**Published:** 2020-11-16

**Authors:** Jingxia Tian, Xiaoying Wei, Weihua Zhang, Aiguo Xu

**Affiliations:** ^1^Department of Respiratory and Critical Care Medicine, First People’s Hospital of Shangqiu, Shangqiu, China; ^2^Department of Nephropathy of Rheumatology, First People’s Hospital of Shangqiu, Shangqiu, China; ^3^Department of Respiratory and Critical Care Medicine, First Affiliated Hospital of Zhengzhou University, Zhengzhou, China

**Keywords:** selenium nanoparticle, radiotherapy, combination, lung cancer, synergistic therapy

## Abstract

**Objective:**

To investigate the effects of selenium nanoparticles (nano-Se) combined with radiotherapy on the proliferation, migration, invasion, and apoptosis of non-small cell lung cancer (NSCLC) A549 and NCI-H23 cells.

**Methods:**

Nano-Se was synthesized and characterized by transmission electron microscope (TEM), X-ray diffractometer, and Ultraviolet-visible (UV)-Vis Spectroscopy, separately. The uptake of nano-Se by lung cancer cells was detected by flow cytometry. Cell counting kit-8 (CCK-8) method was used to detect the antiproliferative activity of nano-Se combined with radiotherapy. Wound healing tests and transwell assay were used to detect the migration and invasion ability of the cells. Annexin V-fluorescein isothiocyanate (FITC)/Propidium iodide (PI) staining by flow cytometry was used to detect apoptosis. The expression of Cyclin D1 (CCND1), c-Myc, matrix metalloproteinase 2 (MMP2), MMP9, cleaved Caspase-3, and cleaved Caspase-9 were detected by Western blot.

**Results:**

The average diameter of nano-Se was 24.39 nm and the wavelength of nano-Se increased with the increase of radiation dose under UV-Vis Spectroscopy. The uptake of nano-Se in lung cancer cells was increased with the increase of nano-Se concentration. The nano-Se combined with radiotherapy decreased the proliferation activity of NSCLC cell lines A549 and NCI-H23 in a dose-dependent manner (all *P* < 0.05). Compared with the Control group, nano-Se combined with radiotherapy could significantly inhibit the migration and invasion of lung cancer cells (all *P* < 0.05), and the effects of the combination of nano-Se and radiotherapy was better than that of a single application (all *P* < 0.05). Furthermore, nano-Se combined with radiotherapy could induce apoptosis of lung cancer cells (*P* < 0.05) and nano-Se combined with radiotherapy could significantly inhibit the expression of proliferation-related proteins CCND1, c-Myc, invasion and migration-related proteins MMP2 and MMP9, but conversely promoted the expression of apoptosis-related proteins cleaved caspase-3 and cleaved caspase-9. Conclusion: This study found that nano-Se combined with radiotherapy plays an anti-cancer role in lung cancer cells by inhibiting cell proliferation, migration, and invasion, as well as inducing apoptosis, suggesting that nano-Se may be used as a radiosensitizer in the clinical treatment of lung cancer, but further research is still needed.

## Introduction

Lung cancer is one of the major cancers in the world. Among different types of lung cancer, non-small cell lung cancer (NSCLC) accounts for approximately 85% of the incidence (Duma et al., 2019), 60% of which are diagnosed as advanced, chemotherapy and radiotherapy are the standard treatment of advanced NSCLC (Herbst et al., 2018). In recent years, radiotherapy and chemotherapy have become a recognized tumor treatment model. Compared with radiotherapy alone and chemotherapy or sequential therapy, combined therapy has a better effect on the continuous control of local tumors and the improvement of the cure rate (Pirker, 2020). However, the adverse reactions of radiotherapy and chemotherapy such as bone marrow suppression, nausea, and vomiting hinder its clinical application, and the 5-year survival rate of NSCLC is still 16.1% (Bagcchi, 2017). Therefore, there is an urgent clinical need to find a safer and more effective treatment method. The development of nanomaterials has greatly improved the diagnosis and treatment of tumors (Ding et al., 2019). Traditional anti-tumor drug research often encounters problems such as the lack of specific drug targets and the need for higher doses to achieve higher local concentrations (Zhang et al., 2018). Nanoparticles (NPs), due to their strong permeability and retention effect, are expected to overcome the shortcomings of traditional anti-tumor drugs and are called new anti-cancer drugs (Li et al., 2019). In recent years, many studies have been done around selenium, which is a trace element, because it plays a critical role in health care, including immune response and cancer prevention (Feng et al., 2019). Epidemiological and clinical research results show that selenium can alleviate the hazard of different cancers, for instance, liver cancer, breast cancer, prostate cancer, colon cancer, and lung cancer (Chen et al., 2018). Some synthetic selenium related chemicals, like selenomethionine and methyl selenocysteine, displaying increased anticancer activity with increading doses (Bi et al., 2013; Zeng et al., 2013). As an anti-tumor drug and an essential trace element, selenium has an effective dose approximate to the toxicity level, which greatly limits its usage in clinical treatments (Bhattacharjee et al., 2014). Nevertheless, more and more research have been performed around selenium nanoparticles (Nano-Se) because of their good bioavailability, high biological activity, and low toxicity (Li et al., 2011). According to reports, the toxicity of nano-level selenium (Se0) is lower than that of selenite (Seþ2 or Seþ4) ions, so Nano-Se is anticipated to substitute other kinds of selenium in nutritional supplements or pharmaceutical dosage forms (Wang H. et al., 2007). Similarly, Nano-Se may also be used as a radiosensitizer to improve the effect of radiotherapy and reduce its side effects. Huang et al. (2013) confirmed in breast cancer, liver cancer and other cells that Nano-Se can trigger the overproduction of intracellular peroxides, thereby activating the p53 and MAPKs pathways, and promoting cell apoptosis. In addition, in animal experiments, they found that Nano se suppressed tumor growth via induction of apoptosis mediated by p53. Jiang et al. (2014) also found similar findings, that is, Nano-Se can cause glioma cytotoxicity by activating a variety of apoptosis signaling pathways, thereby exerting an anti-cancer effect. Given the rare research on the effect of Nano-Se on the function of lung cancer cells, this study intends to treat lung cancer cells through the combination of Nano-Se and radiotherapy to observe the changes in cell proliferation, invasion and migration ability, and the impacts of the above treatments on cell apoptosis, to provide new anti-cancer drugs for the clinical treatment of lung cancer.

## Materials and Methods

### Material

Sodium selenite, bovine serum albumin and glutathione were all purchased from Sigma-Aldrich, United States. Human NSCLC A549 and NCI-H23 cell lines were purchased from ATCC, United States, and DMEM, fetal bovine serum, and double antibodies were purchased from Gibco, United States. Cell Counting Kit-8 (CCK-8) kit was purchased from Dojindo Co., Ltd., Japan, and Annexin V-fluorescein isothiocyanate (FITC)/Propidium iodide (PI) apoptosis kit was purchased from Sigma-Aldrich, United States. Strong RIPA protein lysate, protease inhibitor, blocking solution, primary and secondary antibody diluent, ECL protein luminescent solution were all purchased from Shanghai Biyuntian Biotechnology Co., Ltd. Primary antibody: Matrix metalloproteinase 2 (MMP2) (1:1500), MMP9 (1:1500), caspase-3 (1:2000), cleaved Caspase-3 (1:1000), caspase-9 (1:2000), cleaved Caspase-9 (1:1000), Cyclin (Cyclin D1, CCND1, 1:1000), C-myc (1:1500), and β-Actin (1:3000) were purchased from Cell Signaling Technology Company of the United States. Both the Transwell chamber and matrix gel were purchased from Corning Corporation.

### Method

#### Preparation, Characterization and Identification of Nano-Se

A 345.9 mg of sodium selenite powder was dissolved in 200 mL of double-distilled water. After fully dissolving, 40 mL of 10 nM sodium selenite solution and 160 mL of 10 mM glutathione solution containing 200 mg of bovine serum albumin was used to prepare 10 mM sodium selenite stock solution. An appropriate amount of 0.1 M sodium hydroxide was added to adjust the pH value of the sodium selenite stock solution to 7.1, nano-Se and oxidized glutathione can be formed immediately, see reference (Gao et al., 2014) for details. Dialysis of the stock solution with double-distilled water for 72 h (double-distilled water is replaced every 6 h) can separate the oxidized glutathione from nano-Se, and then store the resulting nano-Se solution at 4°C in the refrigerator. A transmission electron microscope (TEM) sample was prepared by dropping a nano-Se suspended particle solution on a carbon-coated copper grid and drying at room temperature (Chen et al., 2015), and a high-resolution TEM (JEOL Ltd., Japan) was used to characterize and observe the synthesized nano-Se and the average particle size was calculated. After counting and measuring more than 100 particles, the average size and particle size distribution of the nano-Se can be determined (Chen et al., 2018). After that, the X-ray diffraction pattern (λ = 0.15419 nm) of the sample was recorded using a Rigaku X-ray diffraction (XRD), and the Ultraviolet-visible (UV)-Vis Spectroscopy (Agilent Technologies, United States) was used to detect the absorbance of nano-Se at 200 to 400 nm under different doses of irradiation in the range. The final concentration of selenium in the aqueous solution was measured using an atomic absorption spectrophotometer (Hitachi, Japan).

#### Cell Culture

The NSCLC cell lines A549 and NCI-H23 were cultured in DMEM medium containing 10% fetal bovine serum and 1% penicillin-streptomycin. The culture conditions of the incubator were 37°C and 5% CO_2_.

#### Detection of Cell Proliferation Activity by CCK-8 Method

Detection of the effects of nano-Se on the proliferation activity of NSCLC cells: NSCLC cell lines A549 and NCI-H23 were seeded in a 96-well plate at a density of 1 × 10^4^ and 0.8 × 10^4^ cells/well, respectively. After incubating for 1 day, different concentrations of nano-Se (0 (Control), 0.25, 0.5, 1, 2, 5, 10, 15, and 20 μg/mL) were used for treatment for 24 h. Then add 10 μL CCK-8 to each well, and continue to incubate for 2 h in a 37°C incubator, and then measure the absorbance (OD) at 450 nm with a spectrophotometer. Set up a blank background group (Blank), that is, wells with only DMEM medium as a control to avoid medium infection OD value. Each group has four multiple holes.

Detection of the effect of radiation treatment on the proliferation activity of NSCLC cells: Based on the selection of an appropriate exposure concentration of nano-Se, the NSCLC cell lines A549 and NCI-H23 were firstly divided into 1 × 10^4^ and 0.8 × 10^4^ cells/well. The cell/well density was seeded in a 96-well plate, cultured for 24 h, and then treated with 1 μg/mL nano-Se for 24 h. Then wash the cells with phosphate-buffered saline (PBS), replace it with a new normal medium, and irradiate 6 MeV-X rays with a linear accelerator. The average dose rate is 200 cGy/min, and the irradiation doses were 0, 2, 4, and 6 Gy. After the irradiation, put the cells back into the incubator and continue culturing for 24 h, and the rest of the detection process is the same as the previous CCK-8 detection method.

The calculation formula for the relative cell proliferation activity is: relative cell proliferation activity (%) = (OD treatment group-OD Blank)/(OD control group-OD Blank) × 100%.

#### Determination of Nano-Se Uptake of Cells by Flow Cytometry

Since A549 is more sensitive to the toxic reaction of Nano-Se exposure, A549 cells were seeded in a six-well plate at a density of 5 × 10^5^ cells/well, and cultured with culture medium containing different concentrations of nano-Se (0, 0.5, and 1 μg/mL) for 24 h. Take gold nano-particles (Gold nano-particles, nano-Au) treated cells as a positive control group (Chang et al., 2017). After the culture, the cells were digested and harvested with trypsin, washed twice with PBS, and then suspended in 1 mL PBS, and tested on flow cytometry to detect the absorption of nanoparticles by cells.

#### Scratch Test to Detect Cell Migration Ability

A549 and NCI-H23 cells were seeded in a six-well plate and cultured for 24 h to 80–90% confluence. Scribe a straight line with a sterile 200 μL pipette tip perpendicular to the bottom of the plate, wash the fallen cells with PBS, replace the culture medium with fresh medium, and use an inverted microscope connected with a real-time image system to take a picture of the mark. Fresh medium containing 1 μg/mL nano-Se was added to Nano-Se group and nano-Se + Radiation group to culture cells for 24 h, an equal volume of sterilized saline were added to Control group and Radiation group to culture cells for 24 h, then Radiation group and nano-Se + Radiation group received a certain dose of radiation treatment. After the treatment, all the groups were placed in the incubator for 24 h and then photographed again. ImageJ software was used to calculate the relative migration amount of cells according to the scratch gap area (Area) of each group of cells at 0 and 48 h. The formula is: relative cell migration activity (%) = (Area treatment group-0 h-Area treatment group-24 h)/Area treatment group-0 h × 100%.

#### Determination of Cell Invasion Ability by Transwell

Place the Transwell chamber with 80 μL of matrix gel dropwise into the wells of the 24-well plate, and incubate for 2 h in the cell incubator. After the cells were processed accordingly (see “Scratch test to detect cell migration ability” for specific operations), trypsin-EDTA was used to digest the cells, and 200 μL of suspension containing 1 × 10^5^ cells were seeded in the upper chamber of Transwell. At the same time, 750 μL medium containing 10% FBS was added to the lower chamber. After culturing the cells at 37°C for 48 h, remove the medium, fix the cells with 1 mL of 4% paraformaldehyde at room temperature for 10 min, then remove the fixative, wash the cells with PBS once, and add 1 mL 0.5% Crystal Violet solution to each well, stained at room temperature for 30 min, and rinse the cells with PBS. Finally, the cells that invaded the Transwell chamber were counted with an optical microscope.

#### Flow Cytometry to Determine the Level of Apoptosis

The treated cells in the six-well plate (see “Scratch test to detect cell migration ability”) were trypsinized, centrifuged at 400 × *g* at 4°C for 5 min, and 500 μL PBS was added to resuspend the cells to wash and centrifuge to collect the cell pellet. Repeat this step 2. After adding 200 μL of binding buffer to the cells, Annexin V-FITC and PI (10 μL each) were added and mixed gently. After incubating at room temperature for 30 min in the dark, 300 μL of binding buffer was added again, and flow cytometry was immediately performed on the computer. Finally, Flowjo software was used to analyze the results.

#### Western Blot to Determine Cell Protein Expression

The cells were washed twice with pre-cooled PBS and lysed on ice with RIPA strong lysis buffer containing protease inhibitors. Centrifuged at 12,000 × *g* at 4°C for 10 min to take the supernatant and the supernatant was transferred to a new centrifuge tube. Using the BCA method to determine the protein concentration, then add 5× protein loading buffer to mix, and heat at 100°C for 10 min to denature the protein. Take 20 μg protein sample for SDS-PAGE gel electrophoresis (First use 60 V constant voltage electrophoresis until the loading buffer enters the separation gel, adjust the voltage to 80 V and run until the loading buffer is close to the bottom edge of the gel and turn off the power), and transfer the separated protein to PVDF membrane (0.29 A constant current transfer membrane for 90 min). The membrane was sealed in 5% skimmed milk at room temperature for 2 h, washed with TBST three times, then the corresponding primary antibody was added and incubated overnight at 4°C. The next day, the primary antibody was removed, the membrane was washed three times with TBST, and HRP-conjugated secondary antibody was used to incubate with the membrane for 1 h at room temperature, and wash the membrane three times with TBST. By dropping the ECL protein luminescent liquid, the protein bands were visualized in the fluorescence imaging system and photographed to record. Finally, ImageJ software was used to perform grayscale analysis of protein bands.

### Statistical Analysis

Each experiment in this study was repeated at least three times independently, and the measurement data were expressed as mean ± standard deviation, and SPSS 19.0 was used for statistical analysis. Dunnett-test was used to compare differences between groups, and one-way analysis of variance was used to compare multiple groups. *P* < 0.05 was regarded as statistically significant.

## Results

### Characterization and Identification of Nano-Se

**FIGURE 1:**
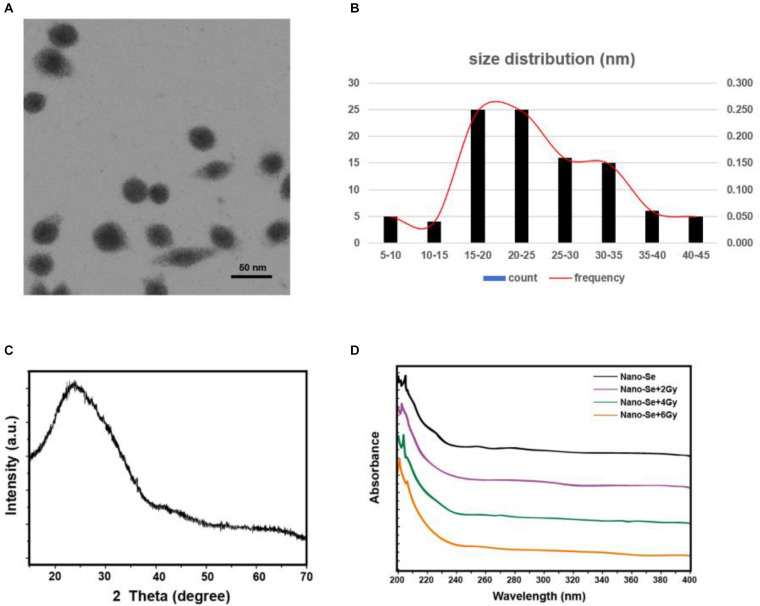
Characterization and identification of Nano-Se. **(A,B)** Observe the morphological size of nano-Se by TEM and draw a size histogram; **(C)** XRD analysis; **(D)** UV-Vis Spectroscopy analysis.

In this study, the synthesized nano-Se was first observed by a TEM, and then the size distribution histogram was drawn, as shown in Figures 1A,B, it can be seen that the average size of nano-Se is 24.39 ± 8.61 nm. In addition, we also performed an XRD analysis. As shown in Figure 1C, no obvious diffraction peaks were found, suggesting that nano-Se is relatively pure. Moreover, as we can see, in Figure 1D, nano-Se showed different absorbance at radiation doses of 0, 2, 4, and 6 Gy, suggesting that the increase in radiation dose may increase the concentration of nano-Se.

### The Effects of Nano-Se and Its Combination With Radiotherapy on Cell Proliferation Activity

**FIGURE 2:**
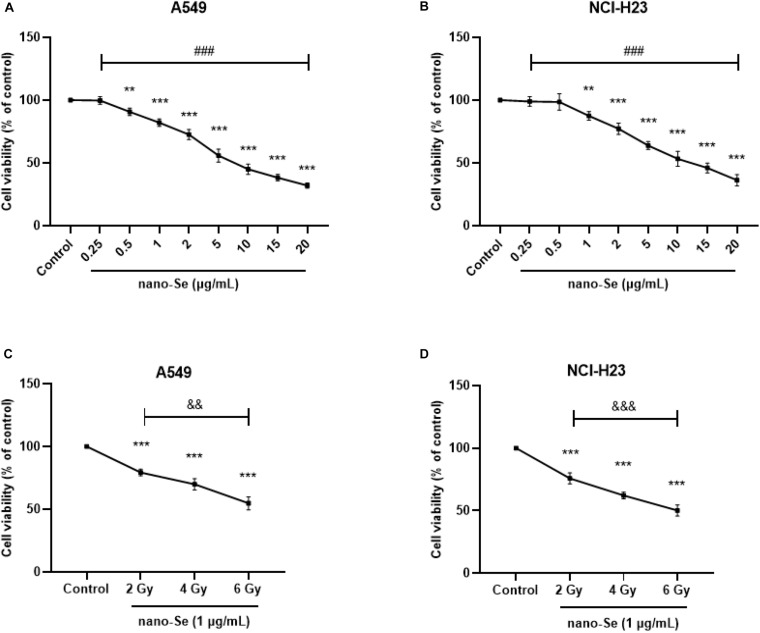
The effect of Nano-Se and its combination with radiotherapy on cell proliferation activity. **(A,B)** The effects of different concentrations of nano-Se (0 (Control), 0.25, 0.5, 1, 2, 5, 10, 15, and 20 μg/mL) on the proliferation of NSCLC cells A549 and NCI-H23; **(C,D)** After 1 μg/mL nano-Se pretreated NSCLC cells A549 and NCI-H23, the effects of different doses of radiation treatment (0 (Control), 2, 4, and 6 Gy) on cell proliferation activity. Compared with the Control group, **P < 0.01, ***P < 0.001; comparison between different concentrations of nano-Se treatment group, ###*P* < 0.001; comparison between different dose radiation treatment groups, &&*P* < 0.01, &&&*P* < 0.001.

As shown in Figures 2A,B, compared with the Control group, treatment of cells with nano-Se can decrease the proliferation activity of lung cancer cells in a dose-dependent manner (all *P* < 0.05), and based on nano-Se exposure to cells, after combined radiotherapy, with the increase of radiotherapy dose, the cell proliferation activity gradually decreased (Figures 2C,D, all *P* < 0.05). Therefore, in subsequent experiments, we chose 1 μg/mL as the exposure concentration of nano-Se and 2 Gy as the radiation dose for radiotherapy.

### Absorption of Nano-Se by NSCLC Cells

**FIGURE 3:**
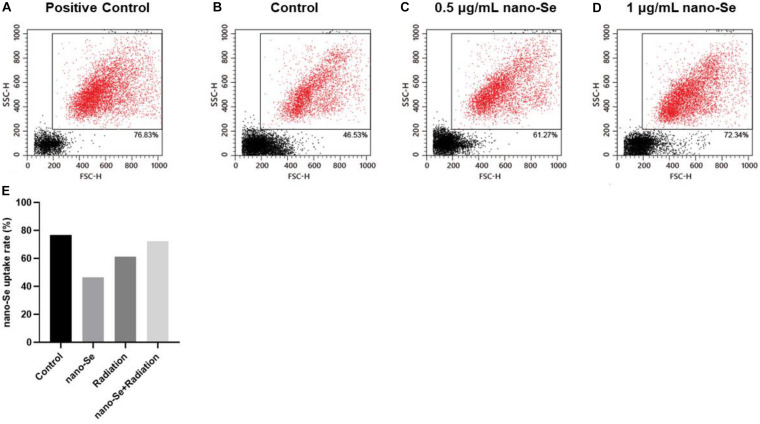
The uptake of Nano-Se by NSCLC cells. **(A–E)** Nano-Au, different concentrations of nano-Se (0, 0.5, and 1 μg/mL) treated A549 cells, the cell absorption of nanoparticles.

As shown in Figure 3, compared with the Control group, as the concentration of nano-Se exposed to NSCLC cells increased, the absorption rate of nano-Se by A549 cells also increased.

### The Effects of Nano-Se Combined With Radiotherapy on Cell Migration

**FIGURE 4:**
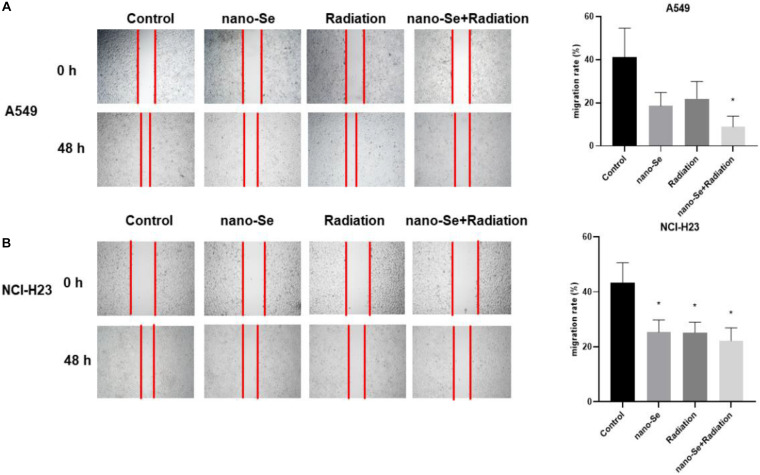
The effects of Nano-Se combined with radiotherapy on cell migration. **(A)** The migration changes of A549 cells; **(B)** The migration changes of NCI-H23 cells. Compared with the Control group, *P < 0.05.

As shown in Figure 4, compared with the Control group, the combination of nano-Se and radiotherapy can significantly inhibit cell migration (*P* < 0.05). The results of A549 and NCI-H23 are consistent. In addition, nano-Se and radiotherapy alone also have a certain inhibitory effect on migration (both *P* < 0.05).

### Effect of Nano-Se Combined With Radiotherapy on Cell Invasion Function

**FIGURE 5:**
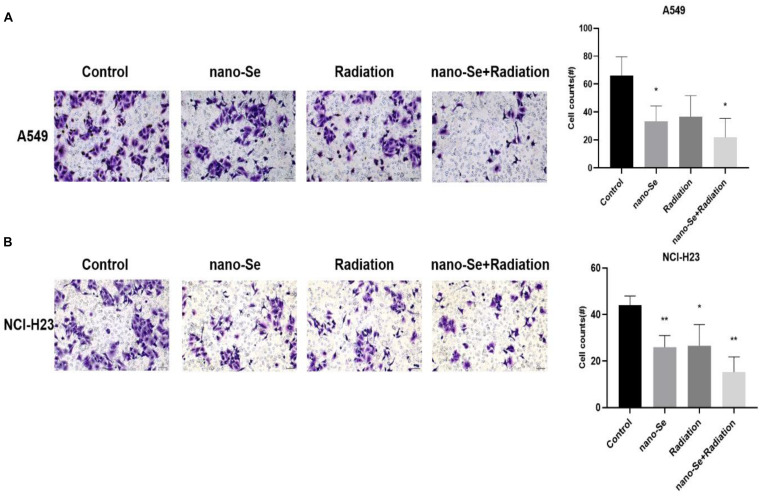
The effects of Nano-Se combined with radiotherapy on cell invasion. **(A)** Invasion of A549 cells; **(B)** Invasion of NCI-H23 cells. Compared with the Control group, *P < 0.05, **P < 0.01.

As shown in Figure 5, compared with the Control group, the combination of nano-Se and radiotherapy can significantly inhibit cell invasion (*P* < 0.05), and nano-Se and radiotherapy alone also have a certain inhibitory effect (both *P* < 0.05).). The results of A549 and NCI-H23 are consistent.

### The Effect of Nano-Se Combined With Radiotherapy on Inducing Cell Apoptosis

**FIGURE 6:**
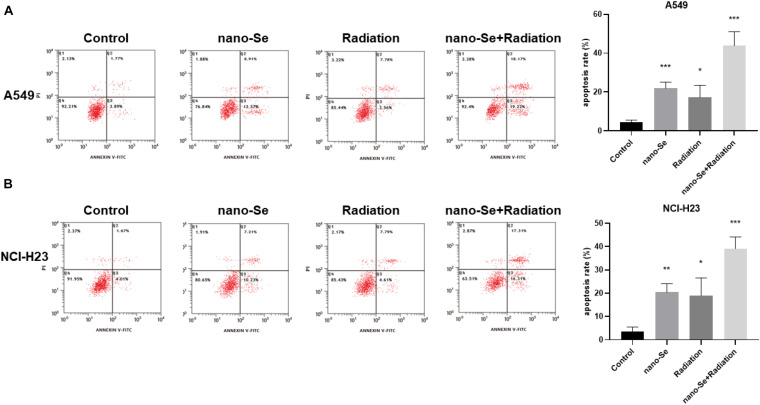
The effects of Nano-Se combined with radiotherapy on cell apoptosis. **(A)** Apoptosis of A549 cells; **(B)** Apoptosis of NCI-H23 cells. Compared with the Control group, *P < 0.05, **P < 0.01, ***P < 0.001.

As shown in Figure 6, compared with the Control group, nano-Se combined with radiotherapy can significantly induce cell apoptosis (*P* < 0.05). The results of A549 and NCI-H23 are consistent.

### The Effects of Nano-Se Combined With Radiotherapy on Cell Proliferation, Invasion, and Migration and Apoptosis-Related Proteins

**FIGURE 7:**
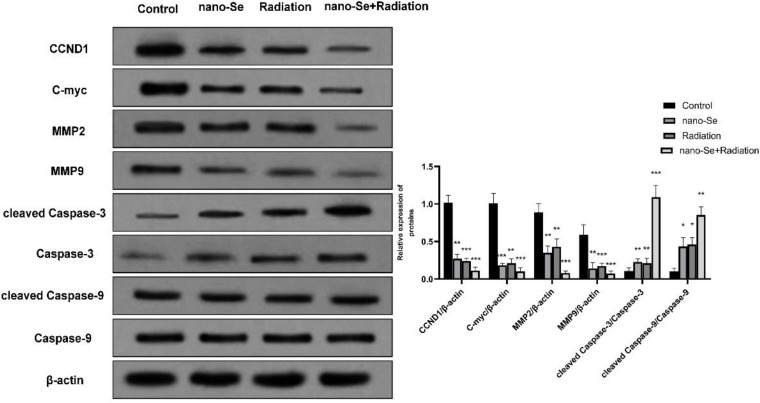
The effects of Nano-Se combined with radiotherapy on the level of protein expression in cells. Compared with the Control group, *P < 0.05, **P < 0.01, ***P < 0.001.

When detecting the protein expression of lung cancer cells, we chose A549 cells for determination. As shown in Figure 7, Western blot detection of protein expression found that compared with the Control group, nano-Se combined with radiotherapy can significantly inhibit the expression of proliferation-related proteins CCND1, c-myc and invasion and proliferation-related proteins MMP2 and MMP9 (both *P* < 0.05), on the contrary, it promoted the expression of apoptosis-related proteins cleaved Caspase-3 and cleaved Caspase-9 (both *P* < 0.05).

## Discussion

Lung cancer, especially NSCLC, is one of the most common cancers in the world, and the desire for clinical exploration of effective treatment methods is very urgent.

Radiotherapy has been widely used in the treatment of lung cancer. Although this method can indeed effectively reduce and eliminate cancerous cells, normal tissues close to the sites where were irradiated will certainly be harmfully affected by radiation. Several radiosensitizers have been produced and appliced to cancer treatment, but the most widely used clinical treatment of lung cancer is still chemotherapy (Man et al., 2010). The ideal way to increase the clinical application of radiotherapy is to find a radiosensitizer that exhibits weak cytotoxicity in the absence of radiation and can effectively promote tumor cell radiation-induced cell death. Selenium is known to have membrane stability and is an indispensable trace elements for animals and humans. Although selenium has a toxic effect when it exceeds a certain level, nano-Se has also been shown to be less poison than inorganic selenium and several organic selenium chemicals (Wang H. L. et al., 2007). It is known that the beneficial effects of Se on the treatment of tumors include various mechanisms, such as reducing DNA damage (Jung et al., 2009), anti-oxidation (Pourkhalili et al., 2012), anti-inflammatory (Miroliaee et al., 2011), enhancing immune response (Ryan-Harshman and Aldoori, 2005), and changing the DNA methylation of tumor suppressor genes state (Yu et al., 2007), inducing apoptosis (Zhang et al., 2013) and blocking the transmission of protein signaling pathways (Christensen et al., 2007).

In this research, nano-Se was first synthesized, and then the particle size of nano-Se was observed by TEM. It was observed by UV spectroscopy that radiation treatment would not destroy the structure of nano-Se, but could enhance the cytotoxicity of nano-Se by increasing the concentration of nano-Se. Also, flow cytometry detected that as the concentration of nano-Se increased, the absorption of nano-Se by cells increased. On this basis, this study used the combination of nano-Se and radiotherapy in NSCLC cancer cells and discussed the effect and mechanism of this combined treatment. First, through the CCK-8 experiment, this study confirmed that the effect of nano-Se combined with radiotherapy on the proliferation of NSCLC cells was greater than that of nano-Se exposure treatment alone or irradiation alone, suggesting that nano-Se and radiotherapy have a synergistic effect in inhibiting cell proliferation activity or to promote each other. Metastasis is a major feature of cancer and the main cause of death of approximately 90% of cancer patients. During this process, the migration and invasion functions of cancer cells play an important role, which also leads to serious consequences of tumor metastasis and poor prognosis (Lu et al., 2017). Therefore, in addition to testing the impacts of nano-Se and radiotherapy on cell proliferation, this study also observed the effect of the combination of lung cancer cell migration and invasion. Consistent with expectations, nano-Se alone or radiation treatment can inhibit cell migration and invasion to a certain extent, and the combined effect of the two is more obvious. In addition to studying the inhibitory effect of the combination of the two on the biological behavior of cancer cells, this study also found that nano-Se combined with radiotherapy can induce apoptosis in lung cancer cells through flow cytometry. The above results suggest that nano-Se combined with radiotherapy has a significant anti-cancer effect.

**FIGURE 8:**
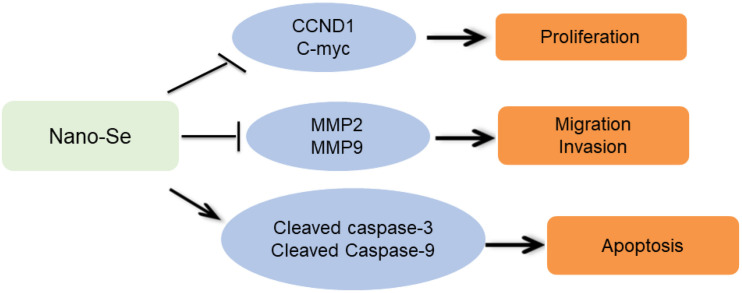
Mechanism pathway diagram of nano-Se acting on NSCLC cells.

Based on the biological functions of nano-Se combined with radiotherapy on lung cancer cells, this study also explored the mechanism, focusing on detecting the expression of several proteins related to cancer cell proliferation, invasion, metastasis, and apoptosis. It is known that CCND1 is an important cell cycle-related protein, and its main function is to regulate the cell cycle and advance the cell from the G1 phase to the S phase (Cai et al., 2014). In tumor cells, CCND1 can accelerate tumor cell division from the G1 phase to the S phase, thereby improving the proliferation ability of tumor cells (Yang et al., 2017); c-Myc is a nuclear protein gene with multiple cell biological functions. It can combine with DNA and chromosomes to regulate cell proliferation (Ericson et al., 2015). Since this study has found that nano-Se combined with radiotherapy can inhibit the proliferation of lung cancer cells through CCK-8 cell proliferation activity experiments, Western blot experiments confirmed that the combination of the two can significantly inhibit the expression levels of CCND1 and c-Myc proteins, further verifying this conclusion. MMPs are a group of zinc-dependent metalloproteinases that regulate a variety of cellular processes, including tumor cell proliferation and metastasis (Egeblad and Werb, 2002). MMP2 and MMP9 are two specific subgroups of MMPs. They have been studied in cancer for many years. Past studies have found that MMP2 and MMP9 were highly expressed in lung cancer and essential for the growth and metastasis of lung tumors (Liu et al., 2019). This study found that the combination of nano-Se and radiotherapy can significantly inhibit the expression of MMP2 and MMP9 proteins by detecting the expression levels of the above two proteins. The results are consistent with the results of Scratch and Transwell. In addition, this study also detected the levels of apoptosis-related classic proteins Caspase-3 and Caspase-9. It is known that Caspase-3 is activated by the proteolytic cleavage of Caspase-9 and is an important apoptosis-executing Caspase. Caspase signal stimulation and accompanying PARP cleavage are considered to be the main features of the apoptotic cascade (Bi et al., 2018), and their levels also indicate the progress of the apoptotic response. In this study, the levels of Caspase-3 and Caspase-9 were detected and found that the expression of cleaved Caspase-3 and Caspase-9 were increased, suggesting the occurrence of apoptosis. This result is consistent with the study of Huang et al. (2013) and Jiang et al. (2014) that nano-Se can induce apoptosis in cancer cells and exert cytotoxicity. The specific mechanism is shown in Figure 8.

## Conclusion

In summary, this study found that nano-Se, as a new form of Se, exerted anti-cancer activity including inhibition proliferation, invasion, migration and promotion of apoptosis of NSCLC cells when combined with radiotherapy. This study provides a theoretical basis for *in vivo* evaluation of the inhibitory effect of nano-Se combined with radiotherapy on lung cancer, but further research is still needed.

## Data Availability Statement

The original contributions presented in the study are included in the article/supplementary material, further inquiries can be directed to the corresponding author.

## Ethics Statement

The animal study was reviewed and approved by the Animal Care and Use Committee at First People’s Hospital of Shangqiu.

## Author Contributions

JT and XW proposed and designed the experiments. JT and WZ drafted the manuscript, interpreted the data, and carried out the experiments with the help of AX and XW. XW and AX revised the manuscript. All authors contributed to the article and approved the submitted version.

## Conflict of Interest

The authors declare that the research was conducted in the absence of any commercial or financial relationships that could be construed as a potential conflict of interest.
